# Surgical Treatment of a Rare Case of Ovarian Fibroma Associated With Elevated CA125 Levels in a Patient of Reproductive Age: A Case Report

**DOI:** 10.7759/cureus.34097

**Published:** 2023-01-23

**Authors:** Anna Thanasa, Efthymia Thanasa, Evangelos Kamaretsos, Ioannis Paraoulakis, Apostolos C Ziogas, Gerasimos Kontogeorgis, Vasiliki Grapsidi, Ektoras-Evangelos Gerokostas, Vasileios Kontochristos, Ioannis Thanasas

**Affiliations:** 1 Department of Anatomy, Department of Health Sciences, Medical School, Aristotle University of Thessaloniki, Thessaloniki, GRC; 2 Department of Histology, Department of Health Sciences, Medical School, Aristotle University of Thessaloniki, Thessaloniki, GRC; 3 Department of Obstetrics and Gynecology, General Hospital of Trikala, Trikala, GRC; 4 Department of Obstetrics and Gynecology, University of Thessaly, Larissa, GRC

**Keywords:** ovarian fibroma, ca125, magnetic resonance imaging, diagnosis, management, case report

## Abstract

Small ovarian fibromas (< 10cm) associated with elevated serum CA125 levels are rarely encountered, particularly in women of reproductive age. We report a rare case diagnosed in a 35-year-old patient after adnexectomy for a solid ovarian mass of approximately 5cm in maximum diameter, accompanied by elevated serum CA125 levels. In preoperative evaluation, no signs of inflammation from the genital tract were found, and no medical history of endometriosis, uterine leiomyomas, or non-gynecological cancer was reported. Intraoperative frozen section biopsy of surgical specimen obtained from the ovarian tumor had negative evaluation for malignancy. Histological examination of the surgical specimen confirmed the diagnosis of ovarian fibroma. The postoperative course was uneventful. Two months after surgery, the blood serum CA125 levels were within normal ranges. The patient is assessed at regular intervals in the gynecology outpatient clinic. In this paper, based on the data of the modern literature, a brief review of this rare nosological entity is made.

## Introduction

Ovarian fibromas are tumors originating from the connective tissue of the ovarian cortex and include three pathological subtypes: fibroma, thecoma, and fibrothecoma [1]. Ovarian fibromas were first described by Young and Scully in 1983 [2]. Fibromas are the most common benign solid tumors of the ovaries and are usually diagnosed in perimenopausal and postmenopausal women between their fifth and sixth decades of life. In most cases the tumors are unilateral, rarely they occur bilaterally, and it is estimated that they concern 1% - 4% of all ovarian tumors [3,4]. These tumors are mostly asymptomatic and are diagnosed very late. In some cases, they may undergo torsion and manifest clinically with acute surgical abdomen [5], while in other cases, they may be associated with ascites and pleural effusion, as in Meigs syndrome [6]. Ovarian fibroma with elevated blood serum CA125 levels is rarely encountered in clinical practice and is very likely to be misdiagnosed as epithelial ovarian carcinoma, particularly when it concerns menopausal patients [7].

The present case report highlights the significant difficulties related to the diagnosis and therapeutic approach of ovarian fibromas associated with elevated serum CA125 levels, especially in those patients who are of reproductive age and wish to preserve fertility and achieve future pregnancy. At the same time, it is pointed out that the knowledge of the clinical, laboratory and imaging characteristics of the ovarian fibroma associated with elevated CA125 levels is considered essential in up-to-date daily clinical practice. This has the effect of facilitating accurate preoperative diagnosis and selecting the most appropriate surgical treatment for this unusual ovarian tumor.

## Case presentation

**Figure 1:**
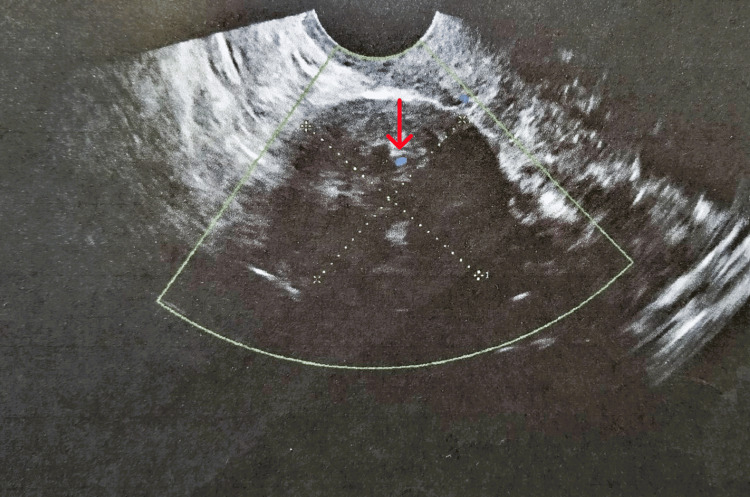
Transvaginal ultrasound and Doppler ultrasound imaging of an ovarian fibroma (our case) A solid hypoechoic mass with clear margins and minimal Doppler flow signals (red arrow) are typical ultrasound features of ovarian fibroma

The case report concerns a 35-year-old patient, with two vaginal deliveries in her obstetric history, who came to the gynecological outpatient clinic for a routine gynecological check-up. From the personal medical history, apart from hyperlipidemia, no other morbidity was reported. The patient was not pregnant, no medical history of endometriosis, uterine leiomyomas, or history of non-gynecological cancer was reported. The hereditary history was free. The clinical examination revealed no signs of pelvic inflammatory disease. Transabdominal ultrasound did not reveal the presence of ascites, but this type of medical imaging was not particularly diagnostic regarding imaging the pelvic organs. Transvaginal ultrasound showed a solid, discrete, echogenic mass occupying the anatomic position of the right ovary, with no space-occupying lesion from the uterine corpus (Figure 1).

**Figure 2:**
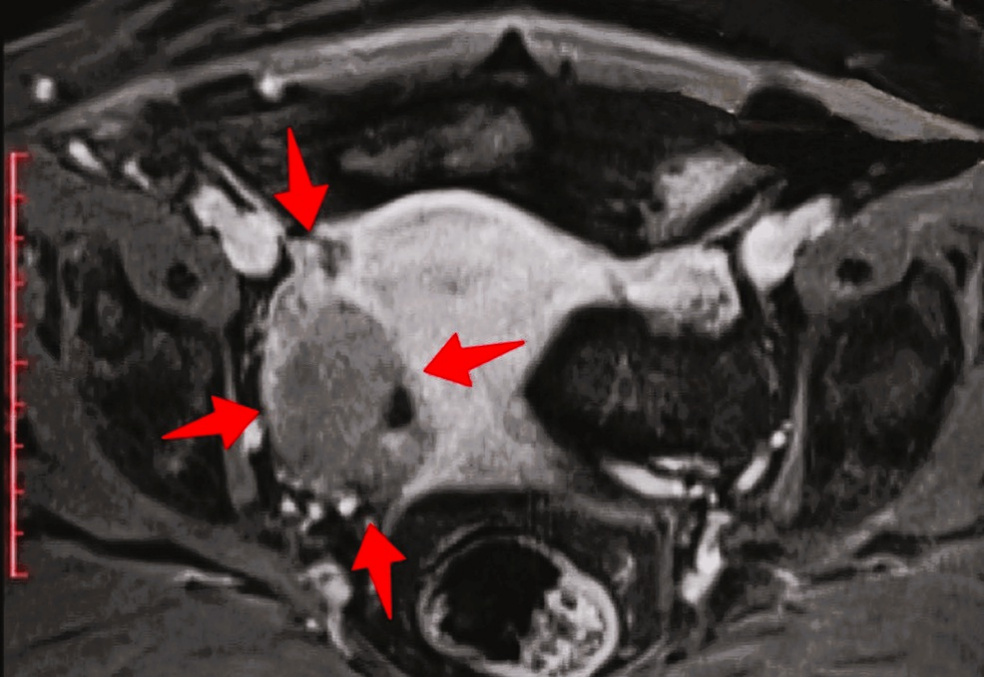
MRI of ovarian fibroma (our case) In the anatomical position of the right adnexa (red arrows) the presence of a pathologic space-occupying lesion that occupies the ipsilateral ovary is highlighted, without the presence of imaging of normal ovarian tissue

Magnetic resonance imaging confirmed the ultrasound findings, but could not rule out ovarian malignancy or pedunculated subserosal fibroid of the uterine corpus. In the anatomical location of the right ovary, the presence of a multilobular pathological space-occupying mass of low signal intensity was found on T2 sequences, with estimated size of 41x35x42 mm (Figure 2).

Blood serum CA125 levels were elevated (311 U/ml). On the admission of the patient to our clinic, laboratory analysis found: hematocrit (Ht) 42.9%, hemoglobin (Hb) 14.1 gr/dl, platelets (PLT) 218x103/ml, white blood cells (WBC) 9.56x103/ml, neutrophils (NEUT) 55.2%, c-reactive protein (CRP) 0.8 mg/dl. Urinalysis was normal. Chest X-ray and echocardiogram were without abnormal findings.

**Figure 3:**
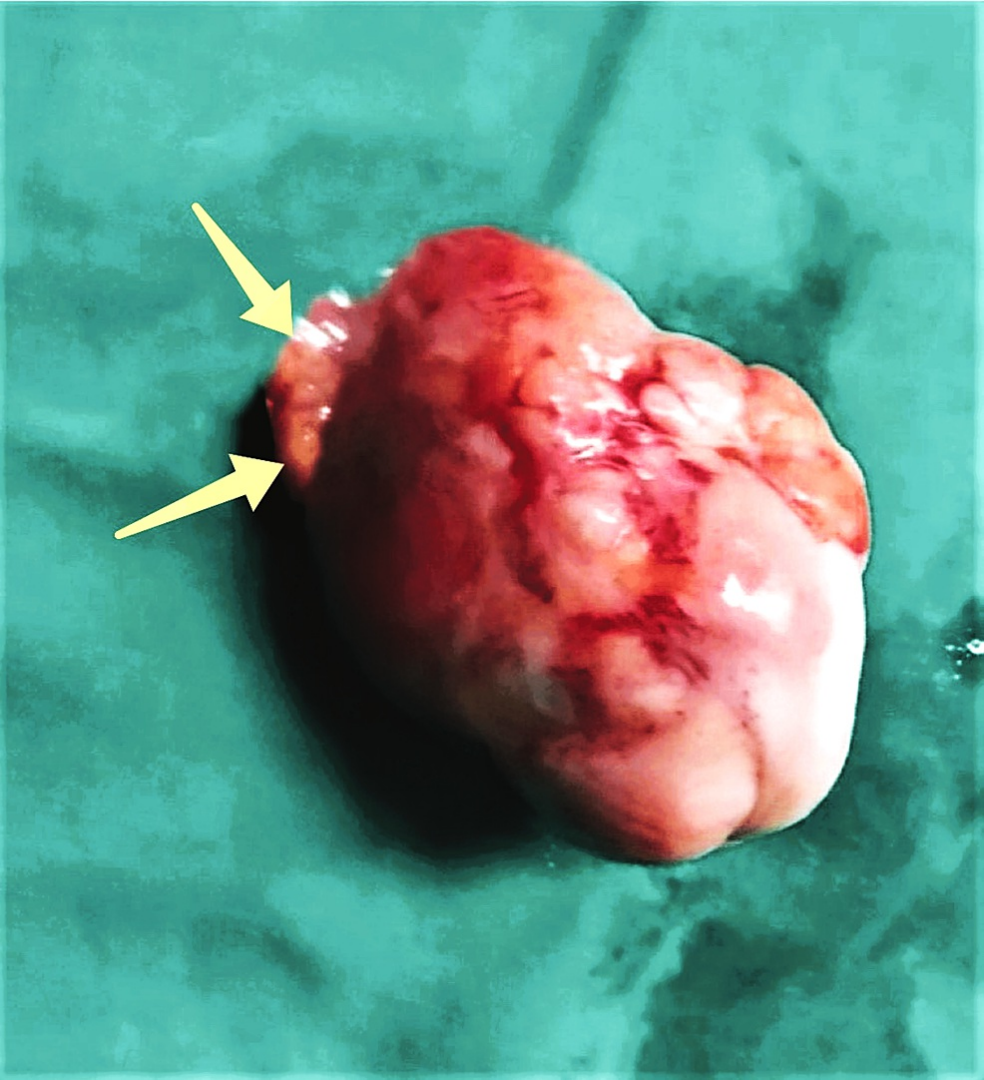
Surgical specimen of ovarian fibroma (our case) The presence of an insignificant area of healthy ovarian tissue (yellow arrows) is characteristic, which led to the decision to surgically resect the fibroma along with the ovary and resect the ipsilateral fallopian tube

After the completion of the pre-operative evaluation, it was decided to treat the patient surgically with laparotomy. Intraoperatively, a solid mass was found in the right ovary, without necrosis and not confluent with the adjacent tissues. The estimated technical surgical difficulties when attempting to exclude the tumor, the increased risk of recurrence and the increased possibility of remaining non-functional ovarian tissue postoperatively led to the decision to remove the ovarian mass along with the ovary (Figure 3) and to remove the ipsilateral fallopian tube at the same time.

**Figure 4:**
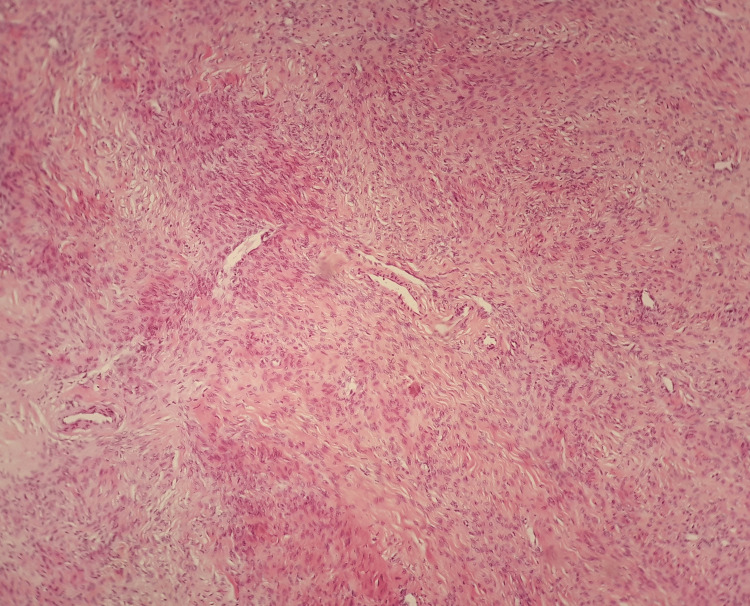
Histological image of ovarian fibroma (our case) A predominantly fascicular pattern of  development of spindle-shaped cells with eosinophilic cytoplasm emerges

**Figure 5:**
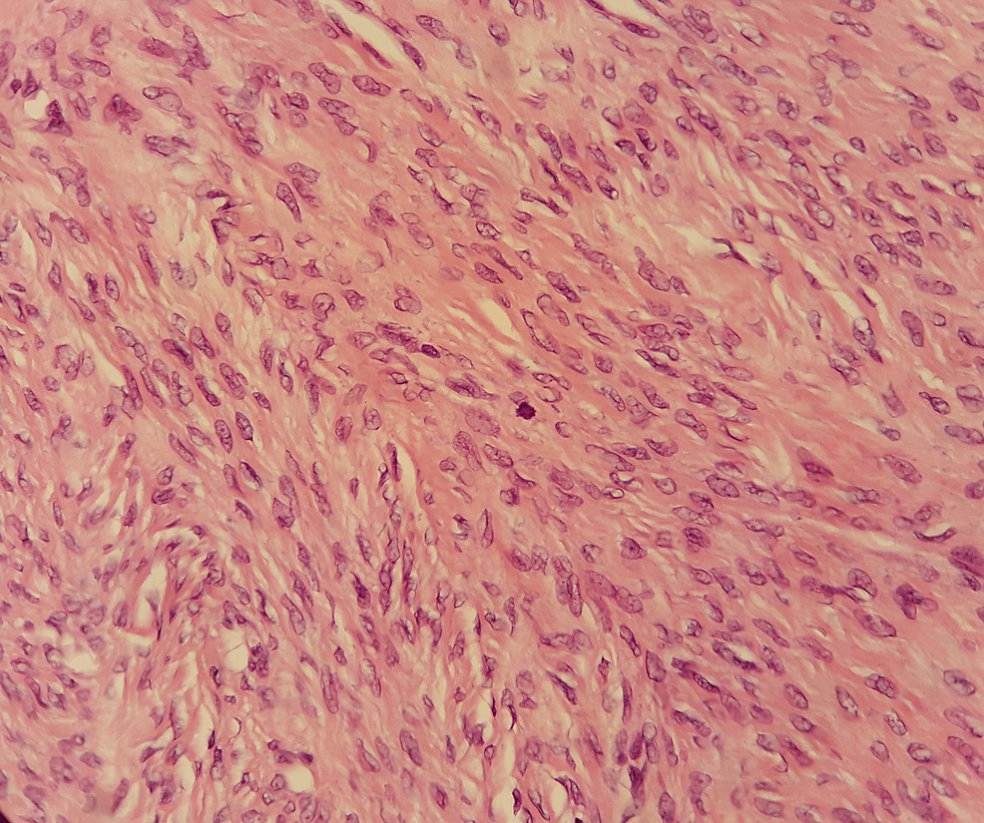
Histological image of ovarian fibroma (our case) On magnification, collagen deposition and mitosis are recognized

Frozen section performed from the tumor intraoperatively was negative for malignancy. Histopathological examination of the surgical specimen confirmed the diagnosis of ovarian fibroma, approximately 45mm in maximum diameter, with mild cellularity and absence of atypia and multiple mitoses (Figures 4, 5).

Cytological analysis of peritoneal washing was negative for malignancy. The postoperative course was smooth, without immediate complications. On the third postoperative day, the patient was discharged from our clinic with recommendation for re-examination at the gynecological outpatient clinic. Two months after surgery, the serum CA125 levels were within normal range. The patient is attended at regular intervals in the gynecology outpatient clinic.

## Discussion

The clinical-laboratory diagnosis of ovarian fibromas is not easy. Although ovarian fibromas are usually asymptomatic and diagnosed randomly, in about half of the cases (43.5%) the main symptom is abdominal pain, which is usually of low intensity [8]. Acute abdominal pain that requires immediate treatment characterizes those cases in which the tumor has undergone torsion and necrosis [9]. The solid nature of ovarian fibromas, their association with ascites and pleural effusion (Meigs syndrome), and elevated serum CA125 levels require further diagnostic investigation to rule out ovarian malignancy [10].

Elevated serum CA125 levels are significantly associated with enlarged ovarian fibromas (diameter ≥ 10cm) or the presence of Meigs syndrome [7]. In our patient, the uncommon condition is that the presence of a small-sized fibroma (maximum diameter < 5cm), without the involvement of ascites or pleural effusion, was associated with increased levels of blood serum CA125. This fact implies significant diagnostic difficulties and serious dilemmas in the management of ovarian neoplasia, including the differential diagnosis of ovarian fibroma from epithelial ovarian carcinoma, despite the young age of our patient. The absence of tumor cells or peritoneal mechanical irritation from the increased intraperitoneal pressure that the large tumor or ascites may cause is unable to clearly elucidate the source of CA125 in our patient [11]. Also, a thorough evaluation of our patient revealed no history of non-gynecological cancer, pelvic inflammatory disease, endometriosis, uterine leiomyomas, or peritoneal, pleural, and pericardial inflammation that could justify the elevation of serum CA125 [11]. The source of serum CA125 in ovarian fibromas/fibrothecomas remains unclear. The earlier theory that peritoneal mesothelial cells may be the source of elevated serum CA125 in ovarian fibromas/fibrothecomas seems to match our patient [12].

In contrast to clinical and laboratory criteria, the contribution of modern imaging methods to the diagnosis of ovarian fibroids is more decisive. Transvaginal ultrasound and Doppler ultrasound imaging of the pelvis are important tools in the preoperative diagnostic management of the disease. Typical sonographic features that support the diagnosis of ovarian fibromas include solid hypoechoic masses with well-demarcated margins and acoustic attenuation as well as minimal Doppler flow signals [1,13]. A recent study showed that the sensitivity and diagnostic accuracy of Doppler ultrasound for the preoperative diagnosis of ovarian stromal tumors are higher compared to those of two-dimensional ultrasound [14]. Similarly, in our patient it is not unexpected that the well-demarcated solid hypoechoic mass with minimal Doppler flow signals as imaged by Doppler ultrasonography strongly suspected the presence of ovarian fibroma/fibrothecoma.

Computed tomography is difficult to distinguish ovarian fibroma from other ovarian masses. Ovarian fibroma scan be imaged in various ways on a CT scan. Typical imaging features are well-demarcated, oval-shaped, unilateral solid tumors, the parenchyma of which shows isodensity, hypointense or isointense signal, and mild to moderate enhancement following contrast-medium injection [3,15]. The presence of ascites and pleural effusion advocates the diagnosis of Meigs syndrome, the differential diagnosis of which from cardiomyopathy can be very challenging [16]. In 2013, Yen et al. showed that ovarian tumor vasculature, such as can be detected by Doppler ultrasound, CT, and MRI, is typical of ovarian fibromas/fibrothicomas. They also published that for adnexal cystic masses, although considered to be of epithelial origin, the possibility of a stromal tumor should not be excluded [17].

Magnetic resonance imaging is a second-line diagnostic method that has significantly contributed to improving the preoperative diagnostic accuracy of ovarian fibromas. Today, magnetic resonance imaging is undoubtedly the most accurate imaging technique in the characterization of ovarian masses [18]. Typical imaging features include low signal intensity on T2 sequences, which reflects the soft tissue spindle cells and intercellular collagen that are abundant in their stroma. However, in cases of enlarged tumors that reflect various degenerative changes, such as cystic degeneration, edematous changes, hemorrhagic infarction or necrosis caused by torsion, high signal intensity on T2 sequences is typical [19]. MRI is estimated that, based on the special features of conventional imaging, it can significantly contribute to the differentiation of fibromas from other ovarian stromal tumors [20]. In contrast, the preoperative differential diagnosis of ovarian fibromas from uterine pedunculated subserosal leiomyomas or malignant ovarian tumors remains difficult and in some cases may be impossible [21].

Treatment of ovarian fibromas is surgery. Despite their benign status, most operations involve open surgical access with resection of the ipsilateral adnexa [22]. Resection of the fibroma with laparotomy or laparoscopy seems to be the appropriate treatment choice in women who wish to preserve fertility. The recurrence rate is estimated to be about 2% of cases [23]. In our patient, adnexectomy was chosen as the most proper treatment option in a woman of reproductive age with an accompanying increase in blood serum CA125 levels, who, according to her admission, has completed her family, but without being able to exclude the possibility of a desire to achieve future pregnancy. The increased possibility of tumor spread in the peritoneal cavity as a result of the increased technical difficulties when trying to exclude the tumor and the increased risk of recurrence of the ovarian lesion, as assessed by the surgical team, due to the inevitable residual fibromal tissue in the ovary, were the main reasons why surgical resection of the ovarian fibroma with preservation of the ipsilateral ovary was not chosen. In addition, the characteristic presence of an insignificant area of healthy ovarian tissue, which would probably be non-functional postoperatively, was another reason that led to the decision to surgically resect the fibroma along with the ovary and, at the same time, resect the ipsilateral fallopian tube.

Despite the advantages of laparoscopic surgery, most surgeons today seem hesitant to use the laparoscopic approach. The varying degrees of technical difficulties during the attempt to exclude the ovarian tumor, which concern almost all cases, and the challenging accurate preoperative diagnosis of ovarian fibromas do not allow safe laparoscopic resection of ovarian fibromas without peritoneal spread [24]. Laparoscopic access of ovarian fibromas should be severely considered in cases of exophytic ovarian tumors in women of reproductive age [22].

## Conclusions

Small ovarian fibromas (< 5cm) associated with elevated serum CA125 levels are extremely rare. The source of CA125 in ovarian fibromas/fibrothecomas remains unclear. Despite the rarity that characterizes them, it is estimated that ovarian fibromas associated with elevated CA125 levels should be included in the differential diagnosis with epithelial ovarian carcinoma not only in postmenopausal women, but also in women of reproductive age. However, in patients of reproductive age, such as our patient, surgical resection of the fibroma or adnexectomy depending on the surgical conditions and capabilities seems to be the appropriate treatment choice, as ovarian neoplasia associated with elevated serum CA125 levels does not indicate necessarily malignant neoplasm.
